# Survival and glycemic control outcomes among patients with coexisting pancreatic cancer and diabetes mellitus

**DOI:** 10.4155/fsoa-2017-0144

**Published:** 2018-02-21

**Authors:** Nina J Karlin, Shailja B Amin, Heidi E Kosiorek, Matthew R Buras, Patricia M Verona, Curtiss B Cook

**Affiliations:** 1 Division of Hematology & Medical Oncology, Mayo Clinic Hospital, Phoenix, AZ 85054, USA; 2 Biostatistics, Mayo Clinic, Scottsdale, AZ 85259, USA; 3 Department of Information Technology, Mayo Clinic Hospital, Phoenix, AZ 85054, USA; 4 Division of Endocrinology, Mayo Clinic, Scottsdale, AZ 85259, USA

**Keywords:** CA 19-9, cancer, endocrinology, glycemic control, outcomes research, pancreas, survival, therapy

## Abstract

**Aim:**

We aimed to determine the effect of diabetes mellitus (DM) on survival in pancreatic cancer and effects of pancreatic cancer on glycemic control in DM.

**Materials & methods:**

Patients with pancreatic cancer from 2007 to 2015, with and without DM, were matched 1:1. We compared characteristics between the groups and assessed 2-year survival with Kaplan–Meier analysis.

**Results:**

In patients with DM, hemoglobin A_1c_ decreased significantly over time (p = 0.01). In survival analysis, 2-year overall survival estimates were 15% (95% CI: 8–24%) for DM patients versus 26% (95% CI: 17–36%) for non-DM patients (p = 0.55). The hazard ratio for matched pairs was 1.15 (95% CI: 0.75–1.77; p = 0.51).

**Conclusion:**

DM did not decrease survival in pancreatic cancer. Pancreatic cancer did not affect glycemic control.

## Lay abstract

The objective of this study was to identify the effect of diabetes mellitus (DM) on survival of patients with pancreatic cancer and to determine whether pancreatic cancer and its treatment affect glycemic control. From an institutional cancer registry, 226 patients with pancreatic cancer were identified and grouped by the presence of DM (n = 113) or absence of DM (n = 113). The groups were matched by age and year of pancreatic cancer diagnosis. Results indicated that DM does not decrease survival and that pancreatic cancer and its treatment do not affect the control of DM.

Pancreatic cancer is the third leading cause of cancer-related deaths in the USA and has a 5-year overall survival (OS) rate of 8% [1]. This statistic has not improved over the past 40 years [2]. Pancreatic cancer is projected to become the second leading cause of cancer-related deaths by 2020 [3,4]. Poor survival is related to the lack of screening and early detection and advanced stage at presentation. Several known risk factors for the development of pancreatic cancer are family history, chronic pancreatitis, tobacco use, high BMI, western dietary pattern and diabetes mellitus (DM) [5]. The proinflammatory milieu of DM is thought to contribute to the initiation and progression of pancreatic cancer [5,6].

One meta-analysis of 88 studies showed a strong association between DM and the development of pancreatic cancer [7]. Another meta-analysis of 18 studies suggested that DM worsened survival for patients with pancreatic cancer [8]. However, for patients undergoing resection of pancreatic cancer, DM is not thought to negatively affect perioperative outcomes [9]. Some evidence also suggests that DM may be a harbinger of pancreatic cancer. Patients with new-onset DM have an eightfold higher risk of pancreatic cancer than those without DM [2,10]. However, no evidence-based recommendations currently exist for screening patients with DM for pancreatic cancer.

Given the complex interplay between DM and pancreatic cancer, we sought to analyze data from our outpatient oncology practice to better understand how pancreatic cancer might affect glycemic control and how DM might affect pancreatic cancer survival. In contrast to the study noted above [8], we previously showed in patients with pancreatic cancer that those with coexisting DM had better OS than those without DM (hazard ratio: 0.60; 95% CI: 0.44–0.80; p < 0.001) [11]. However, in that analysis, many variables were not available that may have affected the conclusions. Therefore, in this case–control study, we aimed to analyze comprehensive patient data on DM and pancreatic cancer variables to investigate whether DM affected pancreatic cancer survival and whether pancreatic cancer and its treatment affected glycemic control among patients with DM.

## Methods

### Case selection

Institutional review board approval was obtained for this retrospective case–control study. We searched our institutional cancer registry for the medical records of patients with newly diagnosed pancreatic cancer who were seen from 1 January 2007 to 31 December 2015. Data were collected regarding age at pancreatic cancer diagnosis, diagnosis date, race/ethnicity and grade/stage of tumor. We then cross-referenced these data against a list of all patients seen during the same period who had a diagnosis of Type 2 DM (*International Classification of Diseases, Ninth Revision* diagnostic code 250.00) to categorize patients with pancreatic cancer by DM status (with or without DM). We excluded patients who received full or partial treatment at another institution or who had another primary cancer. From this dataset, patients with pancreatic cancer and DM were matched by using a greedy algorithm [12] 1:1 to control patients with pancreatic cancer but no DM. Variables included in the matching algorithm were age, sex and year of pancreatic cancer diagnosis. Year of diagnosis was used as a matching variable to achieve similar follow-up durations. Patients were further excluded if no chart review was conducted for their matched pair or if the patient’s DM status could not be verified from the chart review.

Glucose and hemoglobin A_1c_ (HbA_1c_) values were derived from the laboratory information system. We then reviewed the electronic health record for additional detailed information on pancreatic cancer treatment (surgery, chemotherapy, radiation therapy or targeted therapy) and data related to DM (date of DM diagnosis, type of diabetic therapy and diabetic complications).

### Statistical analysis

Patients with pancreatic cancer, with DM (cases) and without DM (controls), were compared on the basis of patient characteristics and clinical variables. Continuous variables were compared by using paired t-tests; categorical variables were compared by using the McNemar test or Bowker test for symmetry. HbA_1c_ levels during the first year after pancreatic cancer diagnosis were evaluated with a linear mixed model in the DM group only (HbA_1c_ values were unavailable for most patients without DM). Time (days) was considered a fixed effect, and an individual-specific random effect was included. A similar approach was used for modeling glucose values during that year. Fixed effects included days, case or control designation, an interaction term (days × case–control designation) and patient-specific and matched pair-specific random effects. Glycemic control was defined as a mean glucose value less than 126 mg/dl during the year after cancer diagnosis.

OS was defined as the time from pancreatic cancer diagnosis until death of any cause. For OS, patients were considered censored at the last known follow-up date if death was not documented in the health records. Two-year OS was estimated with the Kaplan–Meier method and compared between groups by using the log-rank test. Cox proportional hazards regression was used to assess for effect of DM on OS and included matched pairs as the strata variable. Sample size was based on the number of available cases from 2007 to 2015; it provided 80% power to detect a difference in 2-year survival rate estimate of 10 versus 25% between cases and controls. A p-value <0.05 was considered statistically significant; SAS version 9.4 (SAS Institute, Inc., NC, USA) was used for analysis.

## Results

### Patient characteristics

We initially identified 113 patients with pancreatic cancer and DM during the study period and matched them to 113 control patients with pancreatic cancer but without DM. We then performed chart reviews for these 113 matched pairs. After exclusions because of lack of chart review or inability to verify DM status, 92 matched pairs (n = 184) were included in the analysis. Mean (standard deviation [SD]) age was 69.5 (9.0) years, and most patients were white (92%) or non-Hispanic (47%) (Table 1). The most common histologic subtype was adenocarcinoma (88% [161/184]), and 41% of patients had stage IV disease. All characteristics were similar between the DM and non-DM groups, except that patients with DM had significantly greater BMI than those without DM (p = 0.01) (Table 1). Corticosteroids were taken by 23% of patients without DM and 27% of patients with DM.

**Table 1.** **Patient characteristics.^†^**

**Characteristic**	**Total (n = 184)**	**Group**		**p-value**

		**DM (n = 92)**	**No DM (n = 92)**	
Current age, y	69.5 (9.0)	69.3 (8.9)	69.8 (9.1)	0.08^‡^

Age at PC diagnosis, y	68.3 (9.2)	68.1 (9.1)	68.4 (9.3)	0.21

Men	106 (57.6)	53 (57.6)	53 (57.6)	>0.99^§^

White race	170 (92.4)	86 (93.5)	84 (91.3)	0.68^§^

Ethnicity:				>0.99^§^

– Hispanic	4 (2.2)	2 (2.2)	2 (2.2)	

– Non-Hispanic	86 (46.7)	43 (46.7)	43 (46.7)	

– Unknown	94 (51.1)	47 (51.1)	47 (51.1)	

BMI, kg/m^2^	26.8 (5.4) (n = 181)	27.9 (5.6)	25.7 (5.0) (n = 89)	0.01^‡^

Married at the time of cancer diagnosis	142 (77.2)	68 (73.9)	74 (80.4)	0.40^§^

Payer type at the time of PC diagnosis:				0.40^§^

– Medicare	119 (64.7)	61 (66.3)	58 (63.0)	

– Insurance	55 (29.9)	28 (30.4)	27 (29.3)	

– Self-pay	8 (4.3)	2 (2.2)	6 (6.5)	

– Unknown	2 (1.1)	1 (1.1)	1 (1.1)	

Any alcohol use at the time of PC diagnosis:	(n = 183)	(n = 91)		0.11^§^

– Yes	75 (41.0)	31 (34.1)	44 (47.8)	

– No	107 (58.5)	59 (64.8)	48 (52.2)	

– Unknown	1 (0.5)	1 (1.1)	0 (0.0)	

Smoking status at the time of PC diagnosis:	(n = 183)	(n = 91)		0.33^§^

– Never	81 (44.3)	35 (38.5)	46 (50.0)	

– Former	82 (44.8)	44 (48.4)	38 (41.3)	

– Current	19 (10.4)	11 (12.1)	8 (8.7)	

– Unknown	1 (0.5)	1 (1.1)	0 (0.0)	

Employment status at the time of PC diagnosis:				0.97^§^

– Employed	58 (31.5)	27 (29.3)	31 (33.7)	

– Unemployed	5 (2.7)	2 (2.2)	3 (3.3)	

– Retired	99 (53.8)	52 (56.5)	47 (51.1)	

– Unknown	22 (12.0)	11 (12.0)	11 (12.0)	

Tumor stage:	(n = 180)	(n = 90)	(n = 90)	0.90^§^

– I	13 (7.2)	6 (6.7)	7 (7.8)	

– II	30 (16.7)	16 (17.8)	14 (15.6)	

– III	63 (35.0)	32 (35.6)	31 (34.4)	

– IV	74 (41.1)	36 (40.0)	38 (42.2)	

ECOG PS at the time of PC diagnosis:				0.18^§^

– 0	39 (21.2)	18 (19.6)	21 (22.8)	

– 1	123 (66.8)	62 (67.4)	61 (66.3)	

– 2	12 (6.5)	9 (9.8)	3 (3.3)	

– 3	10 (5.4)	3 (3.3)	7 (7.6)	

Use of corticosteroids:	(n = 177)	(n = 88)	(n = 89)	0.46^§^

– Yes	44 (24.9)	24 (27.3)	20 (22.5)	

– No	133 (75.1)	64 (72.7)	69 (77.5)	

^†^Values are mean (standard deviation) or number of patients (%).

^‡^Paired t-test.

^§^McNemar test or Bowker test for symmetry.

DM: Diabetes mellitus; ECOG PS: Eastern Cooperative Oncology Group performance status; PC: Pancreatic cancer; y: Year.

**Figure 1.**
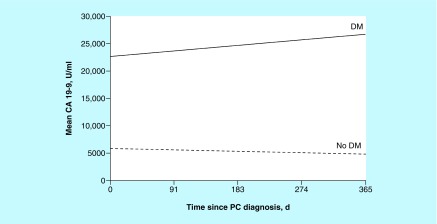
**Estimated mean CA 19-9 value during the year after pancreatic cancer diagnosis.** d: Days; DM: Diabetes mellitus; PC: Pancreatic cancer.

CA 19-9 values (reference range: <37 U/ml) were extremely variable and highly skewed, such that the median (range) CA 19-9 value during the year after diagnosis was 804.2 (3.0–669,280.7) U/ml in the DM group (n = 75) and 394.8 (1.0–173,819.0) U/ml in the non-DM group (n = 78). The mean (SD) values were 24,415.5 (97,052.4) U/ml and 5885.9 (20,585.0) U/ml in the DM and non-DM groups, respectively (p = 0.41) (Figure 1).

### Diabetes mellitus group treatment characteristics

For patients with both pancreatic cancer and DM, the mean (SD) time since DM diagnosis was 8.1 (10.0) years. Most patients were receiving oral agents or insulin at the time of their pancreatic cancer diagnosis (Table 2). Among DM patients, 15 (16%) needed to change their DM therapy within 1 year of pancreatic cancer diagnosis: one patient (7%) switched to diet control as DM therapy, three (20%) switched to oral treatment and 11 (73%) switched to insulin. Overall, 48 patients (52%) were using insulin within 1 year after cancer diagnosis. Insulin use doubled at year 1. DM complications were noted for eight patients (9%) at the time of cancer diagnosis. Among 65 patients (71%) who received chemotherapy, a wide variety of agents were used. In addition, two patients (3%) received targeted therapy and 21 (23%) received radiotherapy. There was no correlation between CA 19-9, HbA_1c_ and glucose values for patients with DM and pancreatic cancer.

**Table 2.** **Diabetes mellitus treatment information in patients with pancreatic cancer.**

**Characteristic**	**Value^†^ (n = 92)**
DM diagnosis preceded PC diagnosis:	68 (74)

– Time since DM diagnosis if preceded PC diagnosis, y	8.1 (10.0)

DM therapy:	

– Diet management	12 (13)

– Oral medication	35 (38)

– Insulin	36 (39)

– Oral medication + insulin	9 (10)

Insulin use at the time of PC diagnosis:	

– Yes	24 (26)

– No	67 (73)

– Unknown	1 (1)

^†^Values are the number of patients (%) or mean (standard deviation).

DM: Diabetes mellitus; PC: Pancreatic cancer; y: Year.

### Pancreatic cancer effect on diabetes mellitus & metabolic control

**Figure 2.**
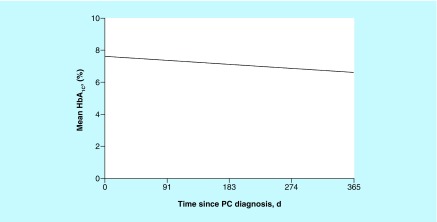
**Estimated mean hemoglobin A_1c_value during the year after pancreatic cancer diagnosis in patients with diabetes mellitus.** d: Days; HbA_1c_: Hemoglobin A_1c_; PC: Pancreatic cancer.

**Figure 3.**
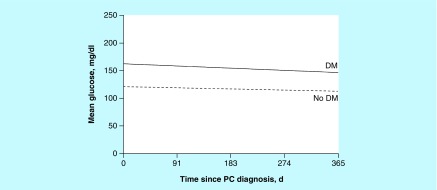
**Estimated mean glucose value during the first year after pancreatic cancer diagnosis.** d: Days; DM: Diabetes mellitus; PC: Pancreatic cancer.

The HbA_1c_ data measured within 1 year after pancreatic cancer diagnosis were available for 57 patients with DM. Of these patients, mean (SD) HbA_1c_ value during the year was 7.3% (1.5%), and 32 (56%) had at least 1 HbA_1c_ measurement of 7.0% or greater (Figure 2). In DM patients, HbA_1c_ significantly decreased over time (p = 0.01). Glucose value during the year after diagnosis among DM patients was significantly higher than among non-DM patients (mean [SD]: 160.6 [38.0] versus 117.2 [19.0]; p < 0.001). Both groups had decreasing glucose values over time (p = 0.008 for time effect) (Figure 3).

### Diabetes mellitus effect on pancreatic cancer survival

**Figure 4.**
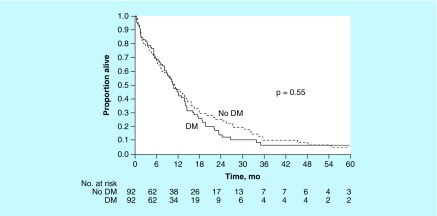
**Kaplan–Meier curves estimating overall survival by diabetes mellitus status.** DM: Diabetes mellitus; mo: Months.

Median OS was 11.0 (95% CI: 9.0–14.1) months for the DM group and 11.2 (95% CI: 8.4–15.8) months for the non-DM group (p = 0.55). With a median (range) follow-up time of 11.9 (0.4–108) months, 2-year OS was estimated at 15% (95% CI: 8–24%) for the DM group and 26% (95% CI: 17–36%) for the non-DM group (p = 0.55) (Figure 4). For the matched pairs, the hazard ratio for death in the DM group was 1.15 (95% CI: 0.75–1.77; p = 0.51). Among patients with available data, median OS for non-DM patients (n = 92) combined with DM patients with good glycemic control (n = 9) was 10.8 (95% CI: 9.2–14.7) months, compared with 11.0 (95% CI: 8.3–14.0) months for DM patients with poor glycemic control (n = 79; p = 0.55).

## Discussion

The study of cancer in the setting of DM is burgeoning, and there is an urgent need for further data on patient-centered outcomes. We previously investigated the effects of several different solid tumors (breast, prostate and lung) and DM on patient outcomes and care [13–15]. In all of these studies, DM did not affect cancer short-term survival, and each cancer did not affect glycemic control in patients with DM.

Applying a matched case–control analysis to investigate how DM affects pancreatic cancer survival and how pancreatic cancer affects metabolic control in DM, we found that DM did not affect OS in pancreatic cancer patients. This result is in contrast to our initial and prior analysis, which showed that in patients with pancreatic cancer, those with coexisting DM had better OS than those without DM [11]. Our previous study did not have a case–control approach and lacked many variables that were available for this more recent study.

In contrast to our findings, a meta-analysis of 18 studies by Shen *et al.* [8] suggested that patients with pancreatic cancer and DM had worse survival than those without DM. We reviewed 16 of these 18 studies and noted that only two were prospective studies. The vast majority of the studies in that meta-analysis were not case–control but  retrospective cohort studies, which are subject to biases (as pointed out by the authors). Because of the nature of the studies included in that meta-analysis, causation – that DM in pancreatic cancer patients is a cause of worse survival – cannot be inferred. The different results of our study may be a factor of the case–control design, which could potentially have an implied causal relationship.

Pancreatic cancer and its treatment also did not adversely affect glycemic control. This is important, because little evidence-based data exist in the literature regarding management strategies for older adults with cancer and comorbid conditions [16]. Among patients with DM, HbA_1c_ significantly decreased over time during the year after diagnosis. However, the mortality rate was so high that it is possible that patients may not have lived long enough to experience worsening glycemic control. Insulin use also doubled at year 1; this is important because more aggressive use of insulin in these patients may be the reason for sustained glycemic control. Furthermore, it is possible that patients may have lost weight after diagnosis, which may have affected glycemic status.

Interestingly, median CA 19-9 value during the year after diagnosis was higher in patients with DM than without DM (804 vs 395 U/ml). Mean CA 19-9 value was also higher in DM (24,415 vs 5886 U/ml), although the difference was not statistically significant (p = 0.41; the analysis was not powered to detect this). Thus, CA 19-9 may not be a reliable marker to help gauge disease progression in patients with DM, and further study is needed. Uygur-Bayramicli *et al.* [17] have postulated that CA 19-9 may represent a marker of pancreatic tissue damage caused by DM. An alternative tumor marker in lieu of CA 19-9 for patients with DM and pancreatic cancer may be needed.

The enzyme ADAR2 (adenosine deaminase that acts on RNA) is important for RNA editing and pancreatic cancer progression. Furthermore, Type 2 DM and other pancreatic diseases are linked to ADAR2 mRNA expression and ADAR2-modulated editing of pancreatic β cells. However, ADAR2 is not a marker currently used in clinical practice in this patient population. Further research is needed with regard to the correlation between pancreatic cancer and pancreatic β cells and ADAR2 [18–21].

There are critical gaps in the literature on patient-centered outcomes for older adults affected by cancer. This was well outlined in a recent Institute of Medicine report by Hurria *et al.* [22]. Further research and evidence-based data are urgently needed to help rectify this age disparity in oncology, such that improved models of care can be developed and ultimately applied to clinical care of elderly patients with cancer and other serious comorbid conditions. This importance is magnified in that the incidence of cancer in persons older than 65 years will increase by 67% from 2010 to 2030 [3]. Although this study was matched for age, it still provides some insight into patient-related outcomes in elderly patients with pancreatic cancer (with and without DM).

This study has some limitations. Although fully powered, sample size still was small and the study duration was short. Ideally, findings should be confirmed in a larger dataset over a longer time. Results of this study most likely have limited applicability to other racial and ethnic groups, because the majority of the full cohort was white. Official causes of death also were not available for this study.

In conclusion, providers and patients can be reassured that DM does not negatively affect survival, and pancreatic cancer and its treatment do not affect glycemic control. Increased CA 19-9 values may be an unreliable tumor marker for gauging disease progression in DM patients with pancreatic cancer. This study is a step toward better understanding the effect of DM on cancer care in the elderly population so that management strategies can ultimately be developed.

## Future perspective

With the findings of this study, providers can be reassured that DM does not affect pancreatic cancer OS and that treatment of pancreatic cancer does not negatively affect glycemic control among patients with DM. Future continued study is needed to address optimal care for patients with these concomitant diagnoses. The increased CA 19-9 levels in patients with DM requires further investigation. This study was not powered to test for differences between the two groups, so additional data are needed to determine whether CA 19-9 is a valid marker in patients with DM.

**Summary points**

- The effect of pancreatic cancer or its treatment on diabetes mellitus (DM) and the effect of DM on pancreatic cancer survival are unknown on an individual level.
- Patients with DM had a significantly higher BMI than those without DM (p = 0.01).
- Among patients with DM, the mean hemoglobin A_1c_ value was 7.3% within 1 year of cancer diagnosis.
- Median overall survival was 11.0 (95% CI: 9.0–14.1) months for the DM group and 11.2 (95% CI: 8.4–15.8) months for the non-DM group (p = 0.55).
- The 2-year overall survival was estimated at 15% (95% CI: 8–24%) for the DM group and 26% (95% CI: 17–36%) for the non-DM group (p = 0.55).
- For the matched pairs, the hazard ratio for death in the DM group was 1.15 (95% CI: 0.75–1.77; p = 0.51).
- In patients with DM, hemoglobin A_1c_ significantly decreased over time (p = 0.01).
- Mean glucose level in the DM group was significantly higher than for patients without DM (p < 0.001). Both groups had decreasing glucose values over time (p = 0.008 for time effect).
